# Identification of *Clonorchis sinensis* in bronchoalveolar lavage fluid and peripheral blood using next-generation sequencing in a patient with septic shock: a case report and literature review

**DOI:** 10.1080/22221751.2025.2511133

**Published:** 2025-05-23

**Authors:** Weipeng Liu, Mengting Su, Dongjing Zhang, Siqi Liu, Yin Zhang, Zhengfei Yang

**Affiliations:** a Department of Emergency Medicine, Sun Yat-sen Memorial Hospital, Sun Yat-sen University, Guangzhou, People’s Republic of China; b Cellular & Molecular Diagnostics Center, Sun Yat-sen Memorial Hospital, Sun Yat-sen University, Guangzhou, People’s Republic of China; c Chinese Atomic Energy Agency Centre of Excellence on Nuclear Technology Applications for Insect Control, Key Laboratory of Tropical Disease Control of the Ministry of Education, Sun Yat-sen University, Guangzhou, People’s Republic of China

**Keywords:** Clonorchis sinensis, next-generation sequencing, pancreatic cancer, ar lavage fluid, periphera blood

## Abstract

We present a case report of a 59-year-old male diagnosed with pancreatic cancer with multiple abdominal metastases, in whom metagenomic next-generation sequencing unexpectedly identified *Clonorchis sinensis* genomic sequences in both bronchoalveolar lavage fluid and peripheral blood specimens. Subsequent examination further confirmed the presence of viable *C. sinensis* eggs in the stool samples. The findings underscore the prognostic significance of enhanced diagnostic protocols for parasitic infections in oncological cases. This investigation demonstrates the clinical utility of molecular diagnostic approaches and alternative biological specimens for detecting *C. sinensis* infection.

## Introduction

*Clonorchis sinensis*, a fish-borne trematode that parasitizes mammalian bile ducts, including in human hosts, causes clonorchiasis endemic to China, Korea, and Vietnam, with global infection estimates reaching 15–20 million individuals [1,2]. This helminthiasis frequently manifests with asymptomatic or subclinical presentations, characterized by nonspecific gastrointestinal symptoms including dyspepsia, nausea, diarrhoea, and abdominal discomfort [3]. Current diagnostic reliance on stool microscopy for egg detection demonstrates limited sensitivity, potentially leading to underdiagnosis and subsequent chronic infection complications. Such chronicity may progress to biliary tract pathologies including obstruction, cholelithiasis, cholangitis, and cholecystitis-established risk factors for cholangiocarcinoma or other gastrointestinal malignancies [4].

The life cycle of *C. sinensis* comprises metacercarial excystation within the duodenal lumen followed by bile chemotaxis-driven migration to biliary microhabitats [2]. Importantly, prior scientific literature contains no documented evidence of *C. sinensis* colonization in haematogenous systems or pulmonary tissues. The present investigation provides unprecedented molecular evidence through comprehensive high-throughput sequencing analysis of bronchoalveolar lavage fluid and peripheral blood samples obtained from a pancreatic cancer patient experiencing concurrent septic shock, establishing the first documentation of *C. sinensis* genetic signatures within these extrahepatic biological compartments.

## Case presentation

A 59-year-old male patient with chronic hepatitis B virus (HBV) infection and hepatic cirrhosis was hospitalized for evaluation of persistent jaundice exceeding two months’ duration. Two months before the admission at the centre of this report, the patient had initially presented with obstructive jaundice at a local medical institution. Diagnostic evaluations revealed cholangiocarcinoma with portal vein infiltration and multiple metastatic lesions in the hepatic hilar and retroperitoneal lymph node regions. The patient underwent sequential endoscopic investigations including gastroscopy, endoscopic ultrasonography (EUS), and endoscopic retrograde cholangiopancreatography (ERCP), culminating in successful biliary stent implantation. Postprocedural management with intravenous cefoperazone–sulbactam (2:1 ratio) antimicrobial therapy achieved a marked reduction in serum bilirubin levels prior to discharge. For definitive histopathological confirmation and advanced oncological care, the patient was subsequently admitted to our tertiary gastroenterology centre. EUS-guided fine needle aspiration (EUS-FNA) biopsy conclusively established the diagnosis of pancreatic cancer, prompting transfer to the Department of Medical Oncology for formulation of comprehensive antitumour therapy.

Upon admission the patient presented with drowsiness, dizziness, fatigue, and abdominal distension accompanied by discomfort. Vital signs revealed hypotension (blood pressure measuring 80/40 mmHg), tachycardia (heart rate of 140 bpm), and adequate oxygenation (SpO₂ of 99% on 2 L/min supplemental oxygen). Laboratory investigations demonstrated leukocytosis (WBC at 15.44 × 10⁹/L with neutrophilic predominance at 88.2%), elevated procalcitonin (18.01 ng/mL), and markedly increased NT-proBNP (1024 pg/mL). Arterial blood gas analysis indicated metabolic acidosis (pH 7.338, lactate 2.5 mmol/L, base excess −4.2 mmol/L) with acute kidney injury evidenced by serum creatinine of 316 μmol/L and urea at 28.3 mmol/L. Hepatic dysfunction parameters included hypoalbuminaemia (24.9 g/L), hyperbilirubinaemia (total bilirubin was 38.6 µmol/L), reduced cholinesterase (768 U/L), and elevated transaminases (AST 101 U/L, ALT 45 U/L).

Contrast-enhanced abdominal CT imaging revealed a solid hepatopancreatic mass radiologically consistent with pancreatic malignancy, demonstrating metastatic involvement of the right adrenal gland, hepatic hilum, and retroperitoneal lymph nodes. Additional findings comprised newly identified peritoneal effusion with inflammatory changes, hepatic cystic lesions, mild intrahepatic biliary dilation, and external biliary stent placement. Thoracic imaging documented multifocal inflammatory infiltrates in the left upper and bilateral lower lung fields, accompanied by minimal pleural effusions and basal subsegmental atelectasis (Supplementary Figure 1).

The patient was admitted to the Emergency Intensive Care Unit from the Oncology Department with presumed septic shock and intra-abdominal infection. Initial management included aggressive intravenous fluid resuscitation, norepinephrine infusion (0.2 µg/kg/min) to maintain haemodynamic stability, methylene blue administration to optimize microcirculatory perfusion, and broad-spectrum antimicrobial coverage with ertapenem (1 g daily). Despite these interventions, progressive metabolic acidosis accompanied by oliguria developed, necessitating the initiation of continuous renal replacement therapy for correction of metabolic derangements and fluid overload. Subsequent neurological deterioration to coma and worsening respiratory failure required endotracheal intubation with mechanical ventilation support. Bronchoscopic alveolar lavage was performed to obtain specimens for conventional microbial culture and targeted next-generation sequencing (tNGS) analysis. While bacterial cultures remained negative during the 7-day observation period, tNGS of bronchoalveolar lavage fluid (Supplementary Figure 2) demonstrated predominant genomic sequences corresponding to *Escherichia coli* and *Klebsiella pneumoniae*, with moderate levels of *C. sinensis* DNA. Parallel blood metagenomic next-generation sequencing（mNGS） analysis (Supplementary Figure 3) confirmed bacteraemia with *E. coli* co-occurring with HBV, Epstein–Barr virus (human herpesvirus 4), and *C. sinensis* sequences. Notably, stool examination identified viable *C. sinensis* eggs in stool specimens (Supplementary Figure 4), though comprehensive analysis of both bronchoalveolar lavage and gastrointestinal drainage fluids failed to detect adult trematodes or ova.

The antimicrobial regimen was modified to ceftazidime–avibactam (1.25 g/q8 h) for targeted antimicrobial coverage, with concurrent administration of praziquantel for *C. sinensis* elimination. Despite these therapeutic interventions, the patient’s septic shock persisted with haemodynamic instability refractory to vasopressor support. Following comprehensive consultation regarding prognosis, the healthcare proxy elected to transition to comfort-focused care. The patient expired on hospital day 21 following withdrawal of life-sustaining therapies.

## Discussion and review of literature

In addition to the current case, our systematic review identified seven case reports and case series in the medical literature documenting 15 instances of pulmonary pathology associated with *C. sinensis* infection (Table 1) [5–11]. Notably, none of these documented cases demonstrated direct observation of *C. sinensis* within lung tissue. Diagnostic confirmation in all reported cases relied solely on the detection of *C. sinensis* eggs in the stool samples.

**Table 1.** Characteristics of cases reported in the literature for pulmonary disease caused by C. sinensis infections.

First Author, year of publication	Patient age, gender	Geographic location	Exposure history	Symptoms	Imaging findings	Diagnosis method	Treatment
Cartwright, 1949[6]	21, male	Shanghai, China	Consumption of “poorly cooked native fish”	Fever, chills, productive cough	Transient bilateral pulmonary infiltrates	*Clonorchis* ova identified in stool	Mapharsen therapy
Engel, 1967[7]	20–50 (9 patients), male	Hong Kong	Unknown	Transitory haemoptysis	Transitory infiltration (seen in two of nine cases)	*Clonorchis* ova identified in stool	No treatment
Mo, 1984[8]	36, male	China	Ingestion of raw freshwater fish and cooked crabs	Epigastric discomfort, fever, diaphoresis	Transient bilateral pulmonary infiltrates	*Clonorchis* ova identified in stool and bile	Praziquantel
Lee, 1998[11]	37, male	Korea	Unknown	Dyspnoea, cough	Nodular pulmonary parenchymal infiltrates	*Clonorchis* ova identified in stool	Praziquantel, corticosteroids
Lee, 2003[9]	54, male	Korea	Ingestion of raw freshwater fish	Rash on extremities	Migrating pulmonary nodules and patchy densities	*Clonorchis* ova identified in stool, positive skin test	Praziquantel
Sheng, 2017[13]	23 months, female	China	Ingestion of raw freshwater crayfish	Cough, wheezing	Bilateral ground-glass attenuation and reticular opacities	Identification of specific IgG in serum	Praziquantel
Reddy, 2021[5]	63, male	China	Ingestion of raw freshwater crayfish	Worsening cough and weight loss	Focal consolidation in the right upper lobe and at the bases	*Clonorchis* ova identified in stool	Prednisone and praziquantel

High-throughput sequencing (HTS) demonstrates superior diagnostic efficacy in pathogen detection through analysis of bronchoalveolar lavage fluid (BALF) specimens obtained from patients with pulmonary infections, especially among critically ill and immunocompromised populations [12]. For critically ill patients where conventional diagnostic methods yield inconclusive pathogen identification results, HTS should be prioritized when the aetiological diagnosis remains indeterminate and may be appropriately implemented at earlier clinical stages to facilitate timely therapeutic interventions. In our case, due to the patient’s requirement for mechanical ventilation and the radiological and clinical progression of pneumonia, BALF was performed. Approximately 10 mL of BALF was obtained for comprehensive pathogen screening. Unexpectedly, targeted next-generation sequencing (tNGS) of the BALF sample revealed genomic sequences of Clonorchis sinensis, representing the first documented identification of this parasite in BALF using this technique. Given the patient’s worsening clinical condition and poor response to empirical antimicrobial therapy, parallel metagenomic NGS (mNGS) was also conducted on approximately 5 mL of peripheral blood, which similarly detected *C.sinensis* sequences. Confirmatory stool examination following these findings evidenced parasitic ovum presence, supporting the hypothesis of pulmonary aspiration secondary to impaired consciousness and subsequent gastroesophageal reflux containing parasite eggs,*Helicobacter pylori* sequences were concurrently identified in the analysis. However, due to the patient’s critical condition, pathological sampling and endoscopic examination of the hepatobiliary system were not performed. Moreover, abdominal imaging did not reveal typical features of biliary parasitic infection, such as bile duct dilatation or intrahepatic calcifications. Alternative potential pulmonary migration routes (adult/larval worm translocation versus portal venous egg dissemination) were deemed biologically implausible based on established parasitic life cycles. We hypothesize that the presence of *C. sinensis* sequences in peripheral blood may be attributed to the entry of parasitic antigens into the bloodstream or hepatic sinusoids, where macrophages, acting as antigen-presenting cells, recognized these antigens and retained residual genetic material. Alternatively, it is possible that during tissue invasion, *C. sinensis* mechanically disrupted or secreted proteolytic enzymes to degrade the small blood vessels within the bile duct wall, thereby facilitating its entry into the circulatory system and subsequent detection of its genetic material in peripheral blood. HTS technology enables sensitive detection of atypical pathogens through a non-culture-based methodology involving human DNA depletion followed by microbial community amplification and sequencing. This approach typically employs bacterial gene-specific or whole-genome analysis, providing comprehensive characterization of microbial community structure, including relative abundance and biodiversity metrics. Importantly, this technique maintains diagnostic utility even in patients with prior broad-spectrum antimicrobial exposure [13].

In 2006, Robertson et al [14] documented a clinical case involving a 21-year-old male presenting with exertional dyspnoea and cough. Microbiological analyses (including bacterial/fungal cultures and specialized staining techniques) of pleural effusion, sputum, BALF and stool samples for common pathogens, acid-fast bacilli, and parasitic elements yielded negative results. The diagnosis of paragonimiasis was ultimately confirmed through positive immunoblot serology for *Paragonimus* species. The patient underwent a 2-day praziquantel regimen (25 mg/kg, three times daily), with subsequent follow-up one month after treatment demonstrating marked clinical improvement. This case highlights the diagnostic challenges in parasitic pulmonary infections, where emerging molecular techniques, such as mNGS of BALF samples, could potentially expedite pathogen identification if contemporaneously available.

Imaging modalities including ultrasound, CT, magnetic resonance imaging, and tissue harmonic imaging constitute essential diagnostic tools for disease detection and progression monitoring [15]. However, these techniques exhibit limited sensitivity and specificity, posing operational challenges for less experienced practitioners. Furthermore the substantial financial investment required for these technologies may restrict their widespread implementation. Recent progress in next-generation sequencing has revolutionized parasitic disease diagnostics by enabling comprehensive multispecies screening. This high-throughput approach facilitates concurrent detection of multiple parasitic organisms within individual clinical specimens, demonstrating considerable potential for advancing parasitological research and clinical diagnostics [16].

In this case, *C sinensis* infection was identified after the diagnosis of pancreatic cancer. The temporal relationship between *C. sinensis* infection and malignancy remains unclear, and it is uncertain whether chronic clonorchiasis contributed to the development of pancreatic cancer in this patient. Notably, the patient had a long-standing history of hepatitis B virus (HBV) infection and liver cirrhosis, both of which are well-established risk factors for gastrointestinal malignancies and may have played a more significant role in tumorigenesis than the parasitic infection itself. However, in regions where human liver fluke infections are endemic, coexisting viral hepatitis is also frequently observed [17]. Studies have demonstrated that individuals coinfected with *C. sinensis* and HBV tend to exhibit more severe liver dysfunction and higher HBV DNA titres compared to those with either infection alone [18]. Therefore, further research is warranted to elucidate the potential interactions between these coinfections and their role in cancer development.

## Conclusion

In conclusion, expedited microbial identification constitutes a critical determinant for optimized clinical outcomes in critically ill patients with infectious complications, while concurrently facilitating the detection of atypical microbial agents. Next-generation sequencing, functioning as a cutting-edge molecular diagnostic modality, empowers clinicians to identify pathogens quickly and accurately in clinical settings, thereby enhancing diagnostic precision and therapeutic decision-making.

## Supplementary Material

Supplementary files_250511.docx
