# Supplementary material for: Identification of Clonorchis sinensis in bronchoalveolar lavage fluid and peripheral blood using next-generation sequencing in a patient with septic shock: a case report and literature review
Source: Emerg Microbes Infect. 2025 May 23;14(1):2511133. doi: 10.1080/22221751.2025.2511133 (PMC12135081; doi:10.1080/22221751.2025.2511133)

**Supplementary Material**

**Supplementary Figure 1.**Chest CT demonstrating multifocal inflammatory infiltrate predominantly localized in the left upper lobe and bilateral lower lobes, accompanied by minimal bilateral pleural effusions and segmental atelectasis in the dependent regions of the lower pulmonary zones.


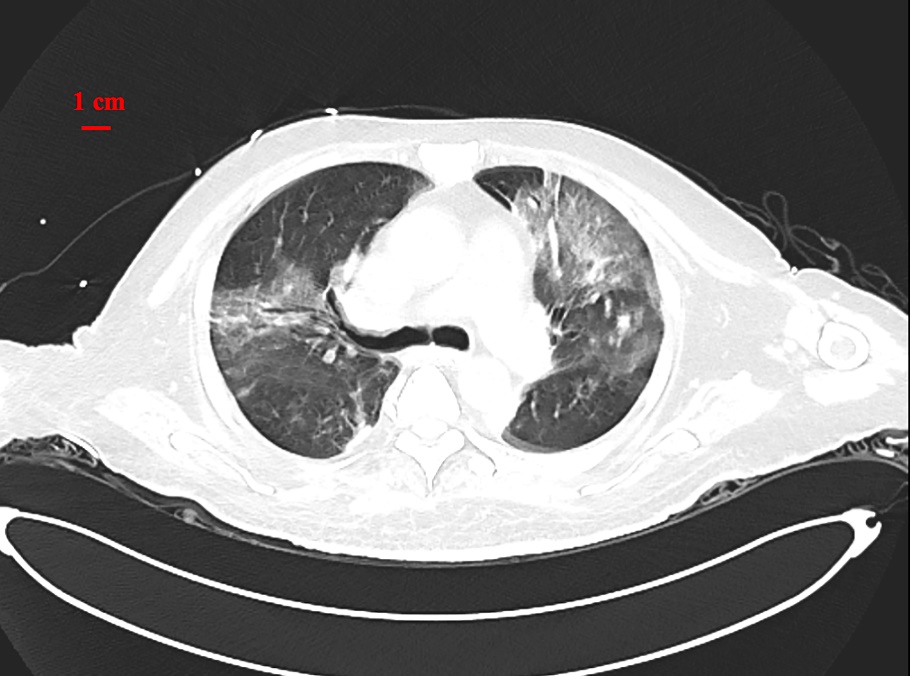


**Supplementary Figure 2.**  Relative abundance of pathogens in bronchoalveolar lavage fluid (BALF) and negative control (NC) through targeted next-generation sequencing (tNGS). The relative abundance of each pathogen was calculated as the log-transformed percentage of the total microbiome composition. Sterile deionized water spiked with a healthy human host fragment was used as the NC, both NC and BALF samples were processed in parallel in each batch. *C. sinensis*: *Clonorchis sinensis*, *K. pneumoniae*: *Klebsiella pneumoniae*, *S. aureus*: *Staphylococcus aureus*, *E. coli*: *Escherichia coli*, *P. acnes*: *Propionibacterium acnes*, *P. stutzeri*: *Pseudomonas stutzeri*, *EVB*: *Human gammaherpesvirus 4*; *TTV*: *Torque teno virus*, *H. pylori*: *Helicobacter pylori*.


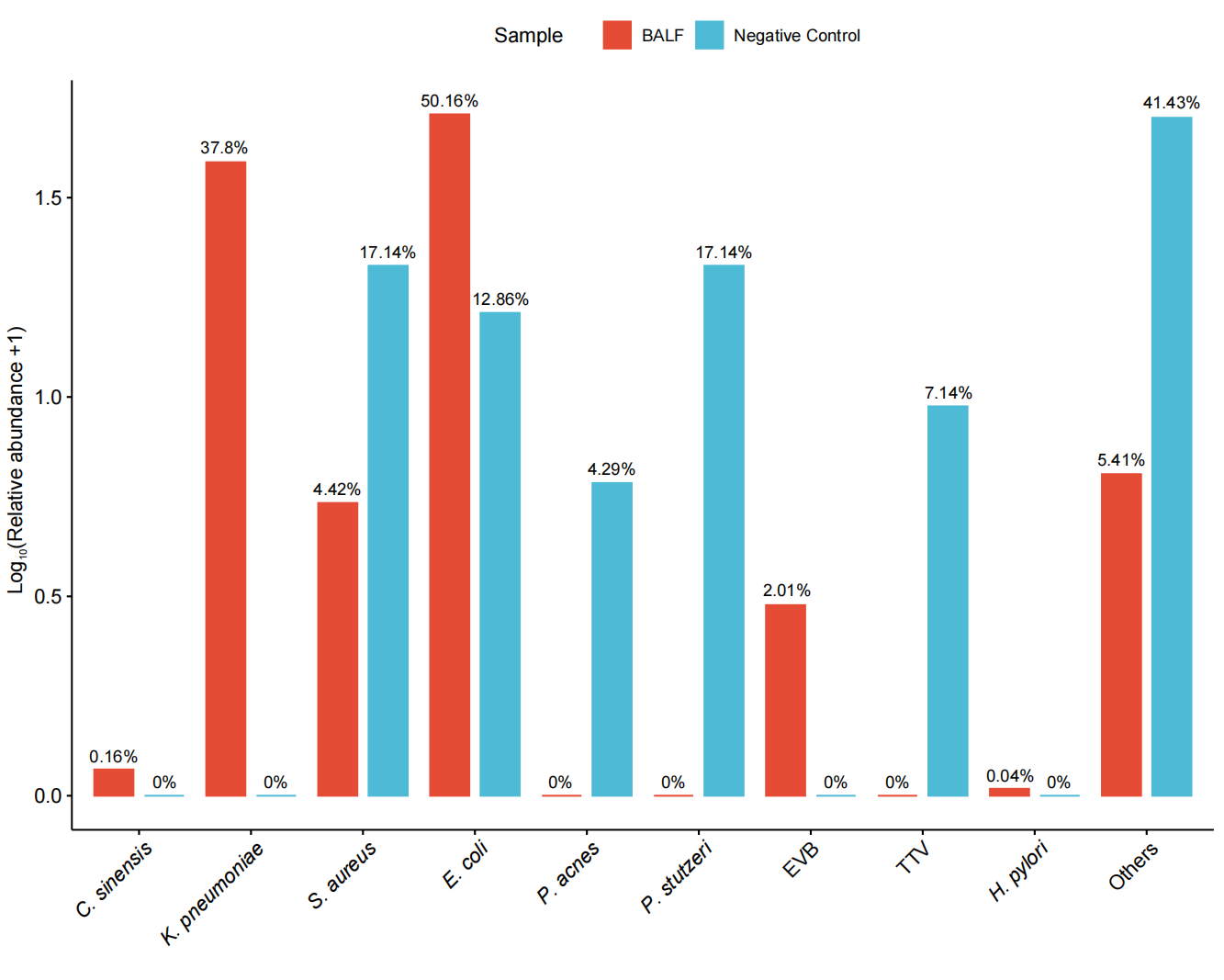


**Supplementary Figure 3.**  Relative abundance of pathogens in plasma and negative control (NC) through metagenomic next-generation sequencing (mNGS). The relative abundance of each pathogen was calculated as the log-transformed percentage of the total microbiome composition. Sterile deionized water spiked with a healthy human host fragment was used as the NC, both NC and plasma samples were processed in parallel in each batch. *C. sinensis*: *Clonorchis sinensis*, *S.epidermidis*: *Staphylococcus epidermidis*, *E. coli*: *Escherichia coli*, *K. pneumoniae*: *Klebsiella pneumoniae*, *EVB*: *Human gammaherpesvirus 4, HBV*: *Hepatitis B virus, TTV*: *Torque teno virus, A.flavus complex*: *Aspergillus flavus complex*.


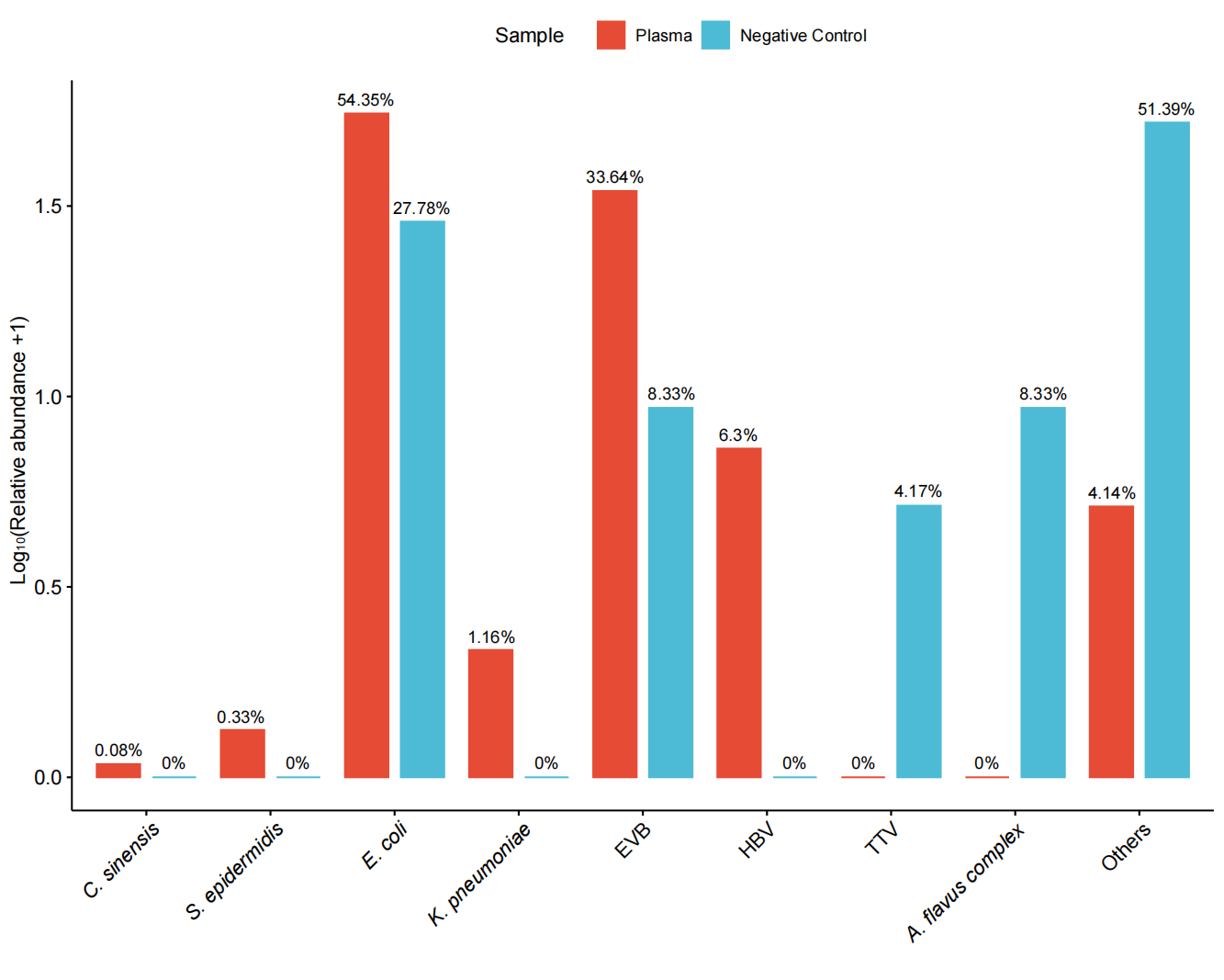


**Supplementary Figures 4.** Detection of *Clonorchis sinensis* egg in the stool sample, with a single oval-shaped ovum, as indicated with the red arrow.


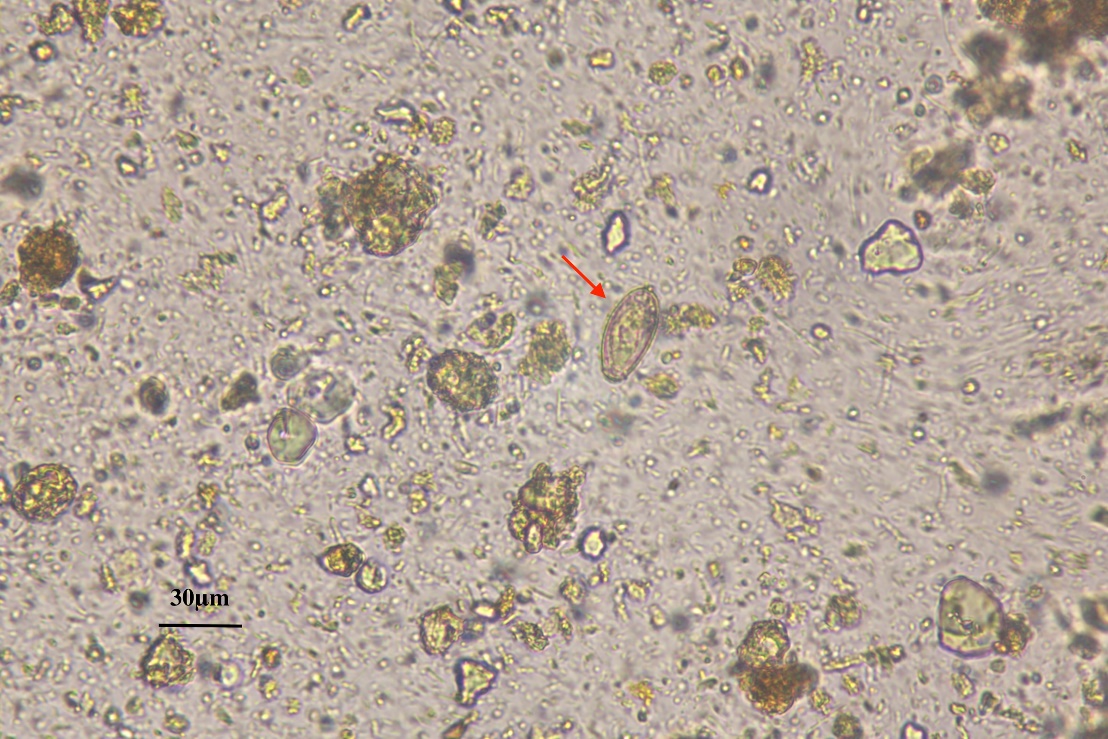

Supplement: Supplementary files_250511.docx [file TEMI_A_2511133_SM1181.docx]
